# Acceptance among the public of weight screening and interventions delivered by dental professionals: observational study

**DOI:** 10.1002/oby.24106

**Published:** 2024-08-20

**Authors:** Jessica F. Large, Andrea Roalfe, Claire Madigan, Amanda J. Daley

**Affiliations:** ^1^ Centre for Lifestyle Medicine and Behavior, School of Sport, Exercise and Health Sciences, National Centre for Sport and Exercise Medicine Loughborough University Loughborough UK

## Abstract

**Objective:**

The objective of this study was to explore the acceptability to the public of receiving weight screening and the offer of support to lose weight from dental teams.

**Methods:**

A cross‐sectional survey was conducted with recruitment of adults from dental practices and community and hospital settings in England and the National Institute for Health and Care Research (NIHR) Be Part of Research initiative.

**Results:**

A total of 3580 participants were recruited across 22 dental sites and the NIHR Be Part of Research initiative. Sixty percent (*n* = 2055/3430) of participants reported that they would be comfortable with their height and weight being measured at a dental appointment. Male participants and those of non‐White race and ethnicity had significantly increased odds of accepting weight screening (odds ratio [OR]: 1.98, 95% CI: 1.66–2.36; OR: 2.07, 95% CI: 1.42–3.03). Fifty‐seven percent (*n* = 1915/3375) of participants reported that it would be acceptable for their dental team to offer support to help with weight management. Male participants and those of non‐White race and ethnicity had significantly increased odds of accepting support (OR: 1.79, 95% CI: 1.49–2.13; OR: 1.62, 95% CI: 1.11–2.37). The most accepted form of support was provision of information on local weight‐management programs (*n* = 1989/2379, 83.6%).

**Conclusions:**

The public is largely receptive to receiving weight screening and the offer of weight interventions from dental teams. Feasibility studies to test the implementation of lifestyle weight interventions in dental settings are required.

**Study Importance**

**What is already known?**

- Weight screening and the offer of support for weight loss are not routinely practiced in dentistry despite dental teams being well placed to deliver brief lifestyle interventions.
- The majority of the public accesses primary dental services (i.e., family dentist) for dental treatment each year; of the limited research to date, most studies have explored the acceptability of weight interventions in secondary care dental settings (i.e., dental hospital).

**What does this study add?**

- Findings suggest that adults are largely supportive of weight screening at a dental consultation and of being offered weight‐management interventions by their dental team.
- Findings suggest that integrating support for weight screening and management at dental consultations could encourage groups, including male individuals and those identifying as non‐White race and ethnicity, to seek support for weight management when they have previously been less likely to do so.

**How might these results change the direction of research or the focus of clinical practice?**

- Findings suggest that it is now important for research to explore how dental teams can integrate screening and support for weight loss within routine care for dental patients.
- Provision of information on local weight‐management programs was the most acceptable form of support and, as such, should be incorporated into future intervention studies.

## INTRODUCTION

Associations between poor dental health and noncommunicable chronic diseases, including cardiovascular disease, type 2 diabetes, and sleep apnea, have been well evidenced [1, 2]. Obesity is a common risk factor for these conditions, and there is an increasing prevalence of children and adults living with overweight or obesity worldwide [3, 4, 5]. Children living with overweight or obesity are more likely to experience dental caries [6]. The Making Every Contact Count (MECC) initiative in the UK requires all health care professionals, including dental teams, to work collaboratively in supporting the public in using opportunistic interactions to support healthy lifestyle changes [2, 7]. Dental teams are suggested to be well placed to provide brief, tailored lifestyle interventions to the public, with 18.1 million adults and 6.4 million children attending a UK National Health Service (NHS) dentist between 2022 and 2023 [8].

The British Society of Pediatric Dentistry supports close liaison with weight‐management services, and the European Federation of Periodontology recommends collaboration between oral health care professionals and doctors, with pathways developed for early detection of noncommunicable diseases in dental practices [2, 9]. Some dental practices (primary dental care) and hospital/community teams (secondary dental care) already engage in weight screening and offer support to patients [1, 10, 11, 12, 13, 14, 15], but this is not routinely offered. Additionally, research has identified that there are several barriers to dental teams offering this type of support [1, 11, 16, 17, 18, 19]. One common barrier is fear of offending patients owing to the sensitive nature of discussions about weight [14, 17, 18, 19]. However, research also suggests that this barrier may be more theoretical, with patient acceptance of discussion of weight and health reported to be favorable [10, 12, 13, 20, 21, 22, 23].

Although promising, research is limited on the public's acceptance of dental teams' delivering of weight interventions. Most studies reporting on public acceptance of weight screening, discussion, or intervention have had small sample sizes [10, 12, 13, 17, 20, 21, 24]. Other studies have been based on data from participants seeking treatment in specialized secondary care settings such as dental hospitals [10, 13, 21]. As most of the public receives dental care from primary dental care services in local communities, it is important to understand support for weight screening and interventions in this context. Therefore, the aims of this observational study were as follows: 1) to investigate whether the public would consider delivery of weight and height screening by dental teams in primary care acceptable; 2) to examine whether the public would find the offer of support for weight loss from dental teams in primary care acceptable; and 3) to determine which forms of support or interventions for weight management the public would find most acceptable. This information will inform future intervention planning and dental health policy.

## METHODS

### Study design and recruitment

This observational study was advertised via Clinical Research Networks in England to dental sites that included dental practices and community and hospital dental services. A total of 22 sites in England were recruited, consisting of 9 NHS or mixed NHS and private dental practices, 2 private dental practices, 5 community dental services, 5 dental hospitals, and 1 dental academy providing primary dental care. Dental sites were responsible for eligibility checks and recruitment. The survey and participant information sheet were available in a digital format using Qualtrics software and on paper when requested by dental practices. The survey was also circulated (by email) via the National Institute for Health and Care Research (NIHR) Be Part of Research (BPoR) initiative, a UK‐wide registry, to adults residing in England, Northern Ireland, and Scotland who volunteer to engage in health care research. Recruitment took place between October 2022 and March 2023. Surveys were completed anonymously. Favorable ethical approval was granted by London, Camden & Kings Cross Research Ethics Committee (reference: 22/PR/0832).

### Eligibility

Sites using online distribution of the survey performed an eligibility search of their patient database. Eligible participants were those aged ≥18 years residing in the UK and able to consent to participate. Eligible participants were then sent a link to the online survey and participant information sheet via email and/or text message to invite them to participate. Reception staff and/or research teams at sites preferring paper distribution of the survey opportunistically invited patients to participate when they attended an appointment. All participating sites had the option to display a standardized study poster in their waiting rooms that contained the online link. Individual online survey links and paper surveys labeled with site codes were used to track responses from sites.

### Input from the public and policy makers

Policy makers and members of the public, including government employees involved in commissioning weight‐management services in England and three dental practices, were consulted during questionnaire design. Feedback was obtained on coherency of structure and adherence to people‐first language to help avoid weight stigma. The survey was piloted within the Centre for Lifestyle Medicine and Behavior (CLiMB) at Loughborough University, Loughborough, UK.

### Study survey

The survey contained seven sections with filtering where relevant, mostly comprising Likert scale questions with some free text questions to explore participant views in more detail. Sociodemographic data were collected, including age range, sex, race and ethnicity, and number of children aged <18 years, as well as dental practice details, last attendance at a dental practice, whether participants considered that they were living with overweight or obesity, and desire to lose weight. Questions explored participants' views on the acceptability of weight screening, weight discussion, and offer of support in dental settings. Views were also sought on how important participants believed dental team involvement in discussing weight and offering support is and whether intervention is a good idea. Caregivers were able to provide demographic information on their child(ren), offer an opinion on the acceptability of weight screening and support for weight management for children, and share any past experiences of weight intervention in dental health settings. See Figure S1 for the study questionnaire.

### Sample size

The sample size was based on the assumption that 50% of participants would find receiving support from their dental team with managing their weight acceptable (yes, definitely agree; yes, probably; and maybe). A minimum of 2400 participants was required to estimate 50% acceptability, with 2% precision at the 95% confidence level.

### Data analyses

Data were analyzed using IBM SPSS Statistics (version 29.0.1.0). Demographic characteristics were summarized using frequencies and percentages. Acceptance of weight screening and acceptance of intervention were analyzed descriptively and summarized using frequencies and percentages. Pearson χ^2^ tests explored the univariable relationships between acceptance of weight screening and support and demographic variables (age, sex, race and ethnicity, dental patient status [NHS vs. private], living with overweight or obesity, and desire to lose weight) presented as percentages and *p* values. Multivariable logistic regression models were used to identify independent demographic variables (as aforementioned) associated with acceptance of weight screening and support with odds ratios (OR), 95% confidence intervals (CI), and *p* values presented. McNemar tests explored whether participants were significantly more likely to accept weight screening over diabetes or cholesterol screening with *p* values presented. Significance was set at *p* < 0.05.

Qualitative data from free text questions were thematically coded. One investigator (Jessica F. Large) performed line‐by‐line coding of participants' perspectives on weight intervention, and views across questionnaires were grouped into similar themes. Two investigators (Jessica F. Large and Amanda J. Daley) reviewed the themes and subthemes using a peer‐debriefing approach to create a framework of analysis.

## RESULTS

### Participants

In total, 3580 questionnaires were completed, with 3463 suitable for analysis; 996 were completed on paper, and 2467 were completed online. Questionnaires returned blank or only containing demographic data (*n* = 17) were excluded. There was no accurate way to identify how many paper questionnaires were distributed, but the overall response rate for the online questionnaire was 13.4% (*n* = 2571). A higher proportion of participants were female (*n* = 2090, 61%), and most respondents identified as White race and ethnicity (*n* = 3177, 92.1%). A total of 294 (8.6%) were aged 18 to 30 years, 408 (11.9%) were aged 31 to 40 years, 510 (14.9%) were aged 41 to 50 years, 810 (23.6%) were aged 51 to 60 years, 919 (26.8%) were aged 61 to 70 years, and 493 (14.4%) were aged ≥71 years. Most participants (*n* = 2925, 85%) reported attending a dental checkup in the past 12 months and seeing an NHS dental provider (*n* = 2328, 67.7%). Nearly one‐third (*n* = 1081, 31.3%) were concerned that they may be living with overweight or obesity, and most participants reported that they would like to lose weight (*n* = 2449, 71.2%). See Table 1 for participant characteristics.

**TABLE 1:** Participant characteristics.

	*n* (%)
Sex (*n* = 3427)	
Male	1322 (38.6)
Female	2090 (61.0)
Intersex	1 (<0.1)
Prefer not to say	14 (0.4)
Age (y; *n* = 3434)	
18–30	294 (8.6)
31–40	408 (11.9)
41–50	510 (14.9)
51–60	810 (23.6)
61–70	919 (26.8)
71+	493 (14.4)
Race and ethnicity (*n* = 3450)	
White^a^	3177 (92.1)
Non‐White^b^	273 (7.9)
Number of children under age 18 y (*n* = 3423)	
0	2608 (76.2)
1	339 (9.9)
≥2	476 (13.9)
Primary dental care service (*n* = 3438)	
NHS	2328 (67.7)
Private	799 (23.2)
Hospital/community service only	39 (1.1)
No dentist	213 (6.2)
Unsure	59 (1.7)
Last dental checkup in primary dental care (*n* = 3441)	
In the past 12 mo	2925 (85.0)
In the past 1–2 y	197 (5.7)
Over 2 y ago	301 (8.7)
Never	18 (0.5)
Living with overweight or obesity (*n* = 3452)	
Yes	1081 (31.3)
No	2156 (62.5)
Unsure	170 (4.9)
Prefer not to say	45 (1.3)
Weight loss wanted (*n* = 3441)	
Yes^c^	2449 (71.2)
No	946 (27.5)
Unsure	46 (1.3)

*Note*: Percentages may not add up to 100% due to rounding. Totals will vary among groups due to difference in response rates among questions.

Abbreviation: NHS, National Health Service.

^a^ White refers to White English/Welsh/Northern Irish/Scottish/British, Irish, and any other White race and ethnicity background.

^b^ Non‐White refers to mixed or multiple race and ethnicity, Asian or Asian British, Black/Black British/Caribbean/African, and other race and ethnicity groups.

^c^ Yes combines “Yes, a lot,” “Yes, a moderate amount,” and “Yes, a little.”

### Weight screening

Sixty percent (*n* = 2055) of participants said that they would “yes, definitely/probably” be comfortable with their height and weight being measured to calculate their body mass index (BMI) at a dental appointment. A further 10.3% (*n* = 354) responded “maybe,” and 28.6% (*n* = 981) were not in favor. From the responses, the three most common reasons given for feeling unsure or not wanting weight and height taken were as follows: “my weight should be discussed by other health care professionals such as my GP [general practitioner]” (*n* = 610), “there are more important dental/health issues to discuss” (*n* = 376), and “I am in good health and do not have weight‐related health problems” (*n* = 257; Table 2).

**TABLE 2:** Participant views on weight screening, weight discussion, and offer of support to lose weight or stop gaining weight provided within a dental setting.

Question	*n* (%)		
Comfortable with weight and height measured to calculate BMI at a dental appointment (*n* = 3430)			
Yes^a^	2055 (60.0)		
Maybe	354 (10.3)		
No^b^	981 (28.6)		
Don't know	40 (1.2)		
Member of dental team willing to discuss weight with			
Dentist (*n* = 3264)	**Yes**	**No**	**Unsure**
	2099 (64.3)	935 (28.6)	230 (7.0)
Dental nurse (*n* = 3245)	**Yes**	**No**	**Unsure**
	2029 (62.5)	995 (30.7)	221 (6.8)
Dental therapist (*n* = 3186)	**Yes**	**No**	**Unsure**
	1773 (55.6)	1101 (34.6)	312 (9.8)
Dental hygienist (*n* = 3186)	**Yes**	**No**	**Unsure**
	1795 (56.3)	1125 (35.3)	266 (8.3)
Receptionist (*n* = 3130)	**Yes**	**No**	**Unsure**
	720 (23.0)	2146 (68.6)	264 (8.4)
Student dentist (*n* = 3180)	**Yes**	**No**	**Unsure**
	1304 (41.0)	1506 (47.4)	370 (11.6)
Non‐dental professional visiting dental practice, i.e., lifestyle coach or nurse (*n* = 3171)	**Yes**	**No**	**Unsure**
	1765 (55.7)	995 (31.4)	411 (13.0)
Acceptable for dental team to offer support to help manage weight (*n* = 3375)			
Yes^a^	1915 (56.7)		
Maybe	493 (14.6)		
No^b^	918 (27.2)		
Don't know	49 (1.5)		
Acceptable forms of support from dental team			
Information about local weight‐management programs (*n* = 2379)	**Yes**	**No**	**Unsure**
	1989 (83.6)	189 (7.9)	201 (8.4)
Referral to local weight‐management programs (*n* = 2343)	**Yes**	**No**	**Unsure**
	1816 (77.5)	262 (11.2)	265 (11.3)
Dental team to ask GP/practice nurse to discuss and support (*n* = 2358)	**Yes**	**No**	**Unsure**
	1918 (81.3)	219 (9.3)	221 (9.4)
Separate appointment at dental practice (*n* = 2322)	**Yes**	**No**	**Unsure**
	1332 (57.4)	553 (23.8)	437 (18.8)
Information about supportive online resources or mobile applications (*n* = 2331)	**Yes**	**No**	**Yes**
	1887 (81.0)	241 (10.3)	203 (8.7)
Acceptable for new weight measurements at future dental appointments (*n* = 3330)			
Yes^a^	2020 (60.7)		
Maybe	306 (9.2)		
No^b^	970 (29.1)		
Don't know	34 (1.0)		
Acceptable for cholesterol check in dental practice (*n* = 3298)			
Yes	2405 (72.9)		
No	685 (20.8)		
Unsure	208 (6.3)		
Acceptable for diabetes check in dental practice (*n* = 3289)			
Yes	2414 (73.4)		
No	680 (20.7)		
Unsure	195 (5.9)		

*Note*: Percentages may not add up to 100% due to rounding. Totals will vary among groups due to difference in response rates among questions. Abbreviation: GP, general practitioner.

^a^ Yes comprises “yes, definitely” and “yes, probably.”

^b^ No comprises “probably not” and “definitely not.”

In the univariable analysis, variables significantly associated with acceptance of weight screening were age (18–30 years, 74.4% vs. 31–40 years, 69.1% vs. 41–50 years, 67.2% vs. 51–60 years, 67.3% vs. 61–70 years, 73.0% vs. 71+ years, 76.4%; *p* = 0.001), sex (female, 65.3% vs. male, 80.1%; *p* = <0.001), race and ethnicity (White, 70.1% vs. non‐White, 82.9%; *p* = <0.001), living with overweight or obesity (no, 72.7% vs. yes, 68.3% vs. unsure, 81.0%; *p* = 0.001), and a desire to lose weight (no, 74.4% vs. yes, 69.7% vs. unsure, 79.5%; *p* = 0.011). Dental patient status (NHS or private) was not significantly associated (NHS, 70.7% vs. private, 69.9%; *p* = 0.675; Table 3). From the multivariable regression analysis (Table 3), adjusting for other variables, age, sex, and race and ethnicity were independently significantly associated with acceptance of weight screening. The odds of acceptance of weight screening increased with age (*p* = 0.004). Male individuals had higher odds of accepting weight screening than female individuals (OR: 1.98, 95% CI: 1.66–2.36). Participants of non‐White race and ethnicity had increased odds of accepting weight screening than participants identifying as White (OR: 2.07, 95% CI: 1.42–3.03).

**TABLE 3:** Univariable and multivariable analysis of participant acceptance of BMI screening and offer of support regarding weight loss/preventing weight gain at dental appointments.

		Univariable analysis χ^2^tests^a^				Multivariable analysis logistic regression models	
Characteristics^b^		Accepting BMI screening,^c^ *n* (%)	*p* value	Accepting support,^c^ *n* (%)	*p* value	Accepting BMI screening, OR (95% CI), *p* value^d^	Accepting support, OR (95% CI), *p* value^d^
Sex	Female	1339 (65.3)	<0.001	1379 (68.3)	<0.001	REF	REF
	Male	1035 (80.1)		993 (78.8)		1.98 (1.66–2.36), <0.001	1.79 (1.49–2.13), <0.001
Age (y)	18–30	212 (74.4)	0.001	230 (81.3)	<0.001	REF	REF
	31–40	273 (69.1)		297 (77.3)		0.81 (0.55–1.19), 0.27	0.89 (0.58–1.37), 0.60
	41–50	334 (67.2)		343 (70.0)		0.70 (0.49–1.02), 0.06	0.54 (0.36–0.81), 0.03
	51–60	535 (67.3)		526 (67.3)		0.74 (0.52–1.05), 0.09	0.50 (0.34–0.72), <0.001
	61–70	664 (73.0)		654 (73.2)		1.03 (0.73–1.46), 0.87	0.71 (0.49–1.04), 0.08
	71+	370 (76.4)		340 (72.3)		1.09 (0.74–1.60), 0.68	0.64 (0.43–0.97), 0.03
	Overall	2388 (71.0)		2390 (72.4)		*p* = 0.004	*p* = <0.001
Race and ethnicity	White	2188 (70.1)	<0.001	2194 (71.7)	0.001	REF	REF
	Non‐White	214 (82.9)		207 (81.8)		2.07 (1.42–3.03), <0.001	1.62 (1.11–2.37), 0.01
Primary dental care service attending	NHS	1608 (70.7)	0.675	1614 (72.3)	0.331	REF	REF
	Private	554 (69.9)		551 (70.5)		0.91 (0.75–1.09), 0.31	0.92 (0.76–1.11), 0.36
Living with overweight or obesity	No	1531 (72.7)	0.001	1499 (72.9)	0.031	REF	REF
	Yes	731 (68.3)		764 (72.2)		0.86 (0.71–1.05), 0.13	1.00 (0.82–1.21), 0.97
	Unsure	132 (81.0)		132 (82.0)		1.34 (0.88–2.06), 0.17	1.48 (0.95–2.30), 0.08
Weight loss wanted	No	690 (74.4)	0.011	654 (73.2)	0.486	REF	REF
	Yes	1679 (69.7)		1720 (72.2)		0.95 (0.77–1.18), 0.65	0.98 (0.79–1.21), 0.84
	Unsure	35 (79.5)		32 (80.0)		1.09 (0.48–2.48), 0.84	1.14 (0.48–2.71), 0.77

Abbreviation: OR, odds ratio.

^a^ Pearson χ^2^ test, significance set at *p* < 0.05.

^b^ Characteristics: Sex: “intersex” and “prefer not to say” were excluded from analysis. Race and ethnicity: White refers to White English/Welsh/Northern Irish/Scottish/British, Irish, and any other White race and ethnicity group. Non‐White refers to mixed or multiple race and ethnicity, Asian or Asian British, Black/Black British/Caribbean/African, and other race and ethnicity groups. Primary dental care service attending: “unsure,” “I do not currently have a dentist,” and “I only visit a hospital or community dental service” were excluded from analysis. Living with overweight or obesity: “prefer not to say” was excluded from analysis. Weight loss wanted: “yes” comprises “yes, a lot,” “yes, a moderate amount,” and “yes, a little.”

^c^ “Accepting BMI screening/support” comprises participants answering “yes, definitely,” “yes, probably,” and “maybe.” No differences in outcomes of significance were found when “maybe” was combined with “no” vs. “yes” answers. Totals will vary among groups due to difference in response rates among questions. Estimates obtained from multivariable regression models.

^d^ OR values > 1 indicate higher odds for acceptance among participants for height and weight screening to calculate BMI than the reference group or higher odds for acceptance among participants for support offered by the dental team than the reference group. REF = reference group with OR = 1.00.

### Discussion and support

Participants expressed a greater preference to discuss weight with a dentist (*n* = 2099, 64.3%) or dental nurse (*n* = 2029, 62.5%) than other dental team members. About half of participants (*n* = 1765, 55.7%) were willing to discuss weight with a non‐dental professional such as a lifestyle coach or nurse (Table 2).

About half of participants (*n* = 1915, 56.7%) would “yes, definitely/probably” find it acceptable for their dental team to offer support to help with managing their weight. A further 493 participants (14.6%) responded “maybe,” and 918 participants (27.2%) would “definitely/probably not” find it acceptable. Characteristics significantly associated with acceptance of support were age (18–30 years, 81.3% vs. 31–40 years, 77.3% vs. 41–50 years, 70.0% vs. 51–60 years, 67.3% vs. 61–70 years, 73.2% vs. 71+ years, 72.3%; *p* = <0.001), sex (female individuals, 68.3% vs. male individuals, 78.8%; *p* = <0.001), race and ethnicity (White, 71.7% vs. non‐White, 81.8%; *p* = 0.001), and living with overweight or obesity (no, 72.9% vs. yes, 72.2% vs. unsure, 82%; *p* = 0.031; Table 3). In the multivariable regression analyses, age remained a significant variable (*p* = <0.001), with participants aged 41 to 50, 51 to 60, and 71+ years having significantly reduced odds of accepting support compared with those aged 18 to 30 years (OR: 0.54, 95% CI: 0.36–0.81; OR: 0.50, 95% CI: 0.34–0.72; OR: 0.64, 95% CI: 0.43–0.97). Male individuals had significantly increased odds of accepting support than female individuals (OR: 1.79, 95% CI: 1.49–2.13). Non‐White participants had significantly increased odds of accepting support than those of White race and ethnicity (OR: 1.62, 95% CI: 1.11–2.37).

The most popular form of support found to be acceptable was for the dental team to provide information on local weight‐management programs (*n* = 1989, 83.6%). Acceptance of the dental team referring to GP offices/practice nurses (*n* = 1918, 81.3%) or to local weight‐management programs (*n* = 1816, 77.5%), as well as information about online resources or mobile applications (*n* = 1887, 81%), was also largely favorable. The least acceptable option was a separate appointment with the dental team to discuss supportive options (*n* = 1332, 57.4%). Free text suggestions from participants included dental teams to collaborate with local gyms; refer to nutritionists, dietetics, and pharmacists; offer telephone or video consultations; and encourage active travel to dental appointments such as with secure bicycle storage and promotion of active lifestyles such as through local park runs.

### Caregiver views

A total of 399/694 (57.5%) caregivers responded “yes, definitely/probably” to being comfortable with their child(ren)'s weight and height being measured at a dental appointment (Table 4). Preferred methods of support to receive from the dental team to help their child(ren) grow into a healthy weight were for the dentist to refer to the child's GP/practice nurse to discuss and offer support (*n* = 448/672, 66.7%), information about online resources or mobile applications (*n* = 413/648, 63.7%), and information about local weight‐management programs (*n* = 417/671, 62.1%). Over half of caregivers would find referral to local weight‐management programs by the dental team acceptable (*n* = 379/662, 57.3%; Table 4).

**TABLE 4:** Participant views on weight screening, discussion, and support offered for their children in a dental setting.

Question	*n* (%)		
Comfortable with child's weight and height measured (*n* = 694)			
Yes^a^	399 (57.5)		
Maybe	85 (12.2)		
No^b^	192 (27.7)		
Don't know	18 (2.6)		
Acceptable forms of support to help children grow into healthy weight			
Information about local weight‐management programs (*n* = 671)	**Yes**	**No**	**Unsure**
	417 (62.1)	178 (26.5)	76 (11.3)
Referral to local weight‐management programs (*n* = 662)	**Yes**	**No**	**Unsure**
	379 (57.3)	198 (29.9)	85 (12.8)
Dental team to ask GP/practice nurse to discuss and support (*n* = 672)	**Yes**	**No**	**Unsure**
	448 (66.7)	158 (23.5)	66 (9.8)
Dental team to ask health visitor/school nurse to discuss and support (*n* = 657)	**Yes**	**No**	**Unsure**
	407 (61.9)	184 (28.0)	66 (10.0)
Separate appointment at dental practice (*n* = 649)	**Yes**	**No**	**Unsure**
	308 (47.5)	254 (39.1)	87 (13.4)
Information about supportive online resources or mobile applications (*n* = 648)	**Yes**	**No**	**Unsure**
	413 (63.7)	171 (26.4)	64 (9.9)
Child's height and weight ever recorded by a dental team (*n* = 698)			
Yes	20 (2.9)		
No	656 (94.0)		
Unsure	22 (3.2)		
Informed by dental team child is living with overweight or obesity (*n* = 697)			
Yes	3 (0.4)		
No	689 (98.9)		
Unsure	5 (0.7)		
Advice or support offered (*n* = 194)			
Yes	3 (1.5)		
No	177 (91.2)		
Unsure	14 (7.2)		

*Note*: Percentages may not add up to 100% due to rounding. Totals will vary among groups due to difference in response rates among questions. Not all participants with parental responsibility for children age <18 years (*n* = 815) responded to the questions.

Abbreviation: GP, general practitioner.

^a^ Yes comprises “yes, definitely” and “yes, probably.”

^b^ No comprises “probably not” and “definitely not.”

The most common reasons (up to three allowed) provided by caregivers for feeling unsure or not wanting their child(ren)'s weight and height taken at a dental appointment included their child being in good health with no weight‐related health problems (*n* = 123), risk of a negative impact on body image and self‐esteem (*n* = 78), and the belief that there were more important dental/health issues to discuss (*n* = 68).

### Cholesterol and diabetes screening

More participants were accepting of diabetes and cholesterol screening than weight screening (paired comparisons diabetes vs. weight: 2385/3053, 78.2% vs. 2211/3053, 72.4%, McNemar test, *p* < 0.001; cholesterol vs. weight: 2376/3050, 77.9% vs. 2211/3050, 72.5%, McNemar test, *p* < 0.001).

### Qualitative data

On review of qualitative data, the three most common themes raised by participants in response to how important dental team involvement in weight intervention is were as follows: 1) scope of practice (*n* = 625, 27.9%); 2) holistic health care (*n* = 264, 11.8%); and 3) duplication of efforts (*n* = 181, 8.1%; Figure S2).

#### Scope of practice

Mixed opinions were shared by participants on whether the dental team should have a role in weight screening and support. Reasons for dental team involvement included a greater opportunity to provide weight interventions and offer longer‐term monitoring and support given the greater frequency of dental checkup appointments in comparison with doctor's appointments. Others proposed that the dental teams' knowledge of diet reinforced the appropriateness of screening and support:

“I believe someone should do it and who better than someone who knows about diet” (participant).

Other participants did not feel it was within the remit of the dental team, but some who shared this view expressed that they would be open to weight screening/support if relevant training had taken place:

“I think it's not really the dentists' place & would prefer to see someone on that field who is experienced” (participant).

“I wouldn't really consider it part of their role but open to it if supported correctly and didn't impact on waiting times, etc.” (participant).

#### Holistic health care

Participants shared views that dental team involvement in weight screening and support would be important from a holistic health care perspective. Collaboration across health care in view of the “huge obesity epidemic” and to “make every contact count” was referred to as grounds for dental involvement. However, some participants did highlight obstacles to the dental team being part of this collaboration.

“I think given the problems with getting a dentist and issues in this country with basic dental health they should really focus on that. However I can see links between dental health and diet, weight, cholesterol, etc., so in an ideal world a holistic approach would be good practice” (participant).

“All medical professionals should be looking at ways that people can prevent future illness‐sometimes it's better not coming from your GP” (participant).

#### Duplication of efforts

Some participants did not perceive support from dental teams to be important in view of weight interventions already being offered by other services, including GP offices, weight‐loss groups, and online resources. However, others proposed that more support may be needed for children. Others expressed the view that weight management is the individual's responsibility and therefore they did not perceive dental input to be important.

“It is an important discussion as part of a healthy lifestyle/better dental health. However, it also duplicates services which should identify concerns and be offered by GP surgeries” (participant).

“Checkups and testing would be great. Not sure how much I personally would use then coming up with a plan vs. managing myself” (participant).

## DISCUSSION

This study explored the acceptability of dental teams offering weight screening and weight‐management interventions to patients. Overall, the concept of these brief interventions taking place in dental settings was viewed positively, suggesting that the public is open to more novel and opportunistic approaches to promote healthier lifestyles as advocated by the NHS, dental stakeholders [2, 7, 9], and the MECC initiative [7]. Male participants and participants identifying as non‐White race and ethnicity were significantly more likely to find weight screening and offer of support acceptable. Participants would prefer to discuss weight with a dentist or dental nurse and would find information on local weight‐management programs the most acceptable intervention. Parents/carers preferred their child(ren) to be referred to their GP/practice nurse to discuss their child's weight and access support.

### Interpretation of findings

Most participants would feel comfortable having their height and weight measured (to calculate BMI) at a dental appointment. Similar levels of support for BMI screening among the public (57.5%–74.2%) were reported in a UK study involving private dental practices [20]. Male sex and increasing age were identified as variables that significantly increased the likelihood of feeling comfortable with weight screening. Male participants were also significantly more likely to accept support. It is possible that these groups may be less self‐conscious of having body measurements taken and thereby more willing to engage in weight screening or feel indifferent to it. Previous research has proposed that male individuals are less likely to see their weight as a problem and experience a reduced frequency of weight stigma, which may explain the greater acceptance to weight screening [25, 26].

In this study, no association was found between those living with overweight or obesity and acceptance of weight screening. However, a multicenter study offering BMI screening across four UK‐based private dental practices reported that participants living with overweight or obesity, although receptive, were significantly more sensitive to BMI screening and receiving healthy weight information in a dental setting or from other health care professionals [20].

Research has shown that weight‐related prejudices are held among some individuals in the dental profession, with lived experience of weight stigma shared by patients, which can be a barrier to weight conversations [16, 17, 27, 28, 29, 30, 31, 32]. In this study, participants expressed a preference for weight discussions to be held with their dentist or dental nurse. This preference may be precipitated by greater familiarity among the public with these team members owing to more frequent interaction with them and/or understanding of their scope of practice. Additionally, more frequent interaction has the potential to foster rapport, which has been proposed as an enabler to successful conversations around weight [13, 31]. However, of note, weight interventions led by dental hygienists and therapists have been reported to be well received by the public, suggesting that use of different members of the team could be acceptable in practice [12].

Most participants agreed that they would find it acceptable or maybe acceptable to be offered weight interventions by their dental team should they wish to lose or stop gaining weight. Male participants and participants of non‐White race and ethnicity were significantly more likely to accept support; however, they make up a much smaller proportion of referrals to weight‐management services [3, 26, 33]. Some of the reasons for possible increased acceptance among male individuals were discussed earlier. Regarding race and ethnicity, research has shown that levels of obesity are higher among some race and ethnicity minority groups, but evidence surrounding referral, engagement, and effectiveness of weight‐management services for race and ethnicity minority groups has been limited [3, 33]. Reasons proposed for reduced access or uptake include: racism; traditional beliefs about food, health, and body image conflicting with mainstream health advice; and lack of culturally appropriate weight‐management services [34, 35]. The increased acceptance of participants identifying as non‐White race and ethnicity to receiving dental‐led weight support highlighted in this study may indicate a more agreeable route to access support for these populations and a way to help reduce inequalities within the population.

Across dentistry, a variety of interventions to support people with their weight have been reported in the literature, including GP referral, goal setting, and signposting/referral to local weight‐management services [10, 11, 12, 15, 19]. In this study, the majority of participants were accepting of referral to local weight‐management programs or GP offices, which is encouraging given that greater weight‐loss outcomes are reported for adults when enrolled in a weight‐management program [36, 37]. The most acceptable intervention was for dental teams to provide information on local weight‐management programs to their patients.

### Strengths and limitations

To our knowledge, this is the first study to assess how acceptable routine weight screening and offer of support from dental teams would be among the public, with recruitment across different regions of the UK and across primary and secondary dental care settings to increase generalizability of findings. The study presents novel insight into preferences for weight discussion and interventions in a dental setting for both adults and children to help guide intervention studies and policy. We have reported quantitative and qualitative research findings to provide wider perspectives on the questions posed.

This study has some limitations, including that participants self‐reported their weight, which is often underreported owing to lack of knowledge about current height and weight or misreporting of information that is accurately known [38, 39]. Moreover, the required sample size for analysis was achieved, but there was a low response rate to the online survey, and study findings may be biased toward members of the public who are more interested in engaging in research or who are more willing to share stronger views. This study focused on reporting the views of the public to hypothetical scenarios, which may be more favorable and supportive than might be the case if participants were directly approached to have their BMI measured when attending a dental appointment. Furthermore, although questionnaire design involved commissioners of weight‐management services, and participants felt able to express their experiences of weight stigma (Figure S2), study design did not involve lived experience.

### Policy recommendations

Dental teams have public support for weight screening and brief interventions but must be supported by their indemnity providers and professional regulators for this to become part of routine practice [1]. Contractual changes must reflect the need to prioritize and provide remuneration for prevention both for oral and general health. These changes would help provide assurances to dental teams that they were working within their scope of practice.

Equally important in supporting dental teams to have discussions around weight is to empower the profession through training and establish clear protocols linking with other health care and weight‐management services. Increasing awareness of weight stigma and increasing clinician confidence could support constructive and sensitive weight discussions and help maintain a healthy clinician‐patient relationship. Incorporating teaching on weight stigma, as well as the links between oral and systemic conditions, into dental undergraduate teaching would be worthwhile. Raising awareness of the interplay between oral and general health among the public may reduce uncertainties over the relevance of dental teams discussing weight and health. Meanwhile, signposting to local weight‐management services such as through posters/flyers within dental practices could be a starting point in response to participants preference for supportive information provision.

## CONCLUSION

Participants were largely supportive of weight screening at a dental appointment and of being offered weight‐management interventions by their dental team. Findings suggest that this more novel approach could encourage groups, including male individuals and people identifying as non‐White race and ethnicity, to seek support for weight management when they have previously been less likely to do so. Conversations with a dentist or dental nurse were preferred. The most popular supportive intervention among adults was to be provided with information on local weight‐management programs, whereas caregivers expressed a stronger preference for their child(ren) to be referred to their GP. These findings highlight important considerations in the design and implementation of future weight interventions in dental settings to assess the feasibility of the wider dental team supporting the public in this collaborative approach to improving health.

## AUTHOR CONTRIBUTIONS

Jessica F. Large was responsible for obtaining ethics approval for the study with input from Amanda J. Daley and Claire Madigan. Jessica F. Large designed the survey, with input from Amanda J. Daley and Claire Madigan, and was responsible for site recruitment and dissemination of the survey. Jessica F. Large collated data and conducted analysis with statistician support (Andrea Roalfe) and support from Amanda J. Daley. Write‐up was completed by Jessica F. Large with review by Amanda J. Daley, Andrea Roalfe, and Claire Madigan.

## FUNDING INFORMATION

Amanda J. Daley is supported by a National Institute for Health Research (NIHR) Research Professorship award (NIHR300026). This research was supported by the NIHR Leicester Biomedical Research Centre. The views expressed are those of the authors and not necessarily those of the National Health Service (NHS), the NIHR, or the Department of Health and Social Care.

## CONFLICT OF INTEREST STATEMENT

The authors declared no conflict of interest.

## Supporting information


**FIGURE S1:** Participant questionnaire.
**FIGURE S2:** Summary table to show common themes shared by participants on the importance of dental teams discussing weight and supporting the public in finding solutions to help the public manage their weight.
**FIGURE S3:** STROBE Checklist.

## Data Availability

All data generated or analyzed during this study are included in this published article (and its online Supporting Information files).
