# Supplementary material for: Acceptance among the public of weight screening and interventions delivered by dental professionals: observational study
Source: Obesity (Silver Spring). 2024 Aug 20;32(12):2364–75. doi: 10.1002/oby.24106 (PMC11589537; doi:10.1002/oby.24106)
Supplement: Supplementary file 1 — FIGURE S1: Participant questionnaire. FIGURE S2: Summary table to show common themes shared by participants on the importance of dental teams discussing weight and supporting the public in finding solutions to help the public manage their weight. FIGURE S3: STROBE Checklist. [file OBY-32-2364-s001.pdf]

Supplementary File

Acceptance amongst the public of weight screening and interventions delivered by dental professionals: Observational study

Jessica F Large*, Andrea Roalfe*, Claire Madigan*, Amanda J Daley*

*Centre for Lifestyle Medicine and Behaviour, School of Sport, Exercise and Health Sciences National Centre for Sport and Exercise Medicine, Loughborough University, UK

Correspondence

Jessica F Large, Centre for Sport and Exercise Medicine, School of Sport, Exercise and Health Sciences, Epinal Way, Loughborough University, Loughborough, LE11 3TU. Telephone: 01509 226372. Email: j.large@lboro.ac.uk ORCID ID: 0000-0002-5092-7088

Contents

Figure S1: Participant questionnaire

Figure S2: Summary table to show common themes shared by participants on the importance of dental teams discussing weight and supporting the public in finding solutions to help the public manage their weight

Figure S3: STROBE Checklist

Figure S1: Participant questionnaire

Your views about dental teams helping people to manage their weight

Welcome to the survey

There are calls for all healthcare staff to work together to promote healthier lifestyle choices, i.e diet and physical activity, and reduce disease in the public. Supporting people with their weight is a key goal of the NHS.

Oral healthcare teams including dentists already discuss and provide advice on diet, alcohol and smoking as part of regular dental care. A few dental practices already offer patients the opportunity to have their cholesterol measured and are checked for diabetes.

We would like to understand your views about whether dental teams should be involved in helping people manage their weight. We would like to know whether you would feel comfortable having your height and weight measured at your dental practice and whether discussing your weight at a dental appointment would be acceptable and helpful to you. If you are a parent or guardian we would also like to hear your views about discussing your child[ren]'s weight with you at your dental practice.

Please now take a moment to read your ***'Participant information sheet'.***

Consent to participate

I voluntarily agree to take part in this study and confirm that I am ≥ 18 years and a UK resident.

(Please tick the box if you wish to take part) ☐

If you would prefer to complete the questionnaire online, please use the link or scan the QR code below:

https://loughboroughssehs.eu.qualtrics.com/jfe/form/SV_9LAR53CWUWV6BLw

Section 1: Some questions about you

1. **What is your age group?**

   Please circle one answer.

   18-30 years 31-40 years 41-50 years 51-60 years 61-70 years 71+ years

2. **Which of the following best describes your sex?**

   Please circle one answer.

   Male Female Intersex Prefer not to say

3. **Which of the following best describes your ethnic origin?**

   Please tick your chosen box.

	Tick
**White**	
English, Welsh, Northern Irish, Scottish or British	
Irish	
Gypsy or Irish traveller	
Roma	
Any other White background	
**Mixed or multiple ethnic groups**	
White and Black Caribbean	
White and Black African	
White and Asian	
Any other mixed or multiple ethnic background	
**Asian or Asian British**	
Indian	
Pakistani	
Bangladeshi	
Chinese	
Any other Asian background	
**Black, Black British, Caribbean or African**	
Caribbean	
African	
Any other Black, Black British or Caribbean background	
**Other ethnic group**	
Arab	
Any other ethnic group	

4. **How many children under 18 years of age do you have parental responsibility for?**

   Please circle one answer.

   0 1 2 3 4 5 >5

5. **Are you registered as an NHS or private dental patient?**

(*This question refers to your local / 'family' / high street dental practice and NOT hospital or community dental services).*

Please circle one answer.

NHS Private

I do not currently have a dentist Unsure

I only visit a hospital or community dental service

If you have answered 'NHS', 'Private' or 'Unsure' please go to Question 6.

If you have answered 'I do not currently have a dentist' or 'I only visit a hospital or community dental service', please go to Question 8.

6. **Please provide us with the name of your dental practice in the box below:**
(If you are unsure, please put 'unsure' and move to the next question).

7. **Please provide us with the city or town your dental practice is found in the box below:**
(If you are unsure, please put 'unsure' and move to the next question).

8. **When was the last time you went to the dentist for a check-up?**

(This question refers to your local / 'family' / high street dental practice and NOT hospital or community dental services).

Please circle one answer.

In the past 12 months In the past 1-2 years Over 2 years ago Never

9. **Are you concerned you may be living with overweight or obesity?**

Please circle one answer.

Yes No Unsure Prefer not to say

10. Would you like to lose weight?

Please circle one answer.

Yes, a lot | Yes, a moderate amount | Yes, a little bit | No | Unsure

Section 2: Weight screening

The following questions ask you about how you would feel having your weight and height taken at the dentist when you visit for a check-up. Measurements would be taken in a private environment and would only involve removing shoes and coats. Weight and height information would help facilitate any healthy lifestyle conversations you wish to have with your dental team.

11) Body mass index (BMI) is a measure that uses your weight and height to work out if your weight is in the healthy zone. Would you feel comfortable with your weight and height being measured to calculate your BMI at a dental appointment?

Please circle one answer.

Yes, definitely | Yes, probably | Maybe

Probably not | Definitely not | Don't know

If you have answered 'Yes, definitely', 'Yes, probably' or 'Maybe' please go to Question 13.

If you have answered 'Probably not', 'Definitely not' or 'Don't know' please go to Question 12.

12) Why would you feel unsure or not want your weight and height taken at a dental appointment?

Please tick up to three answers which best describe why:

	Tick
I am in good health and do not have weight-related health problems	
There are more important dental / health issues to discuss	
I do not feel comfortable / I wish to keep my weight private	
I may be treated differently by the dental team if they know my weight	
There is nothing my dentist can do to help me manage my weight	
My weight should be discussed by another health care professional such as my GP	
Other *(please add suggestions or comments here):*	

Section 3: Weight discussion and support

13) **Which member(s) of the dental team, if any, would you be willing and happy to privately discuss your weight with?**

Please answer 'Yes' 'No' OR 'Unsure' for each option by placing a tick in your chosen box.

	Yes	No	Unsure
**Dentist**			
**Dental nurse**			
**Dental therapist** *(dental professionals who carry out certain items of dental treatment such as fillings)*			
**Dental hygienist** *(dental professionals who help patients maintain their oral health by preventing and treating gum disease and promoting good oral health practice)*			
**Receptionist**			
**Student dentist**			
**Non-dental professional visiting your dental practice such as a lifestyle coach or nurse**			

14) **If you wanted to lose weight or stop gaining weight, would you find it acceptable for your dental team to offer support to help you with managing your weight?**

Please circle one answer.

Yes, definitely   Yes, probably   Maybe

Probably not   Definitely not   Don't know

If you have answered 'Yes, definitely', 'Yes, probably' or 'Maybe' please go to Question 15.

If you have answered 'Probably not', 'Definitely not' or 'Unsure' please go to Question 16.

15) **Would you find it acceptable for your dental team to offer you the following support?**

Please answer 'Yes' 'No' OR 'Unsure' for each option.

	Yes	No	Unsure
Information about weight management programmes in your local area.			
Referral to weight management programmes in your local area with your permission.			
For your dental team to ask your GP / practice nurse to discuss your weight with you and offer support.			
A separate appointment at the dentist to discuss support available.			
Information about online resources or mobile applications (apps) that could support you.			

If you have any suggestions of your own, please add them here:

16) **Would it be acceptable for the dental team to take new weight measurements at future dental check-ups to see if your weight has changed over time?**

Please circle one answer.

Yes, definitely   Yes, probably   Maybe

Probably not   Definitely not   Don't know

Section 4: Cholesterol and Diabetes

If you are living with overweight or obesity and / or have high cholesterol your risk of heart disease increases. Living with overweight or obesity also increases your risk of diabetes. Some dental practices offer free testing to their patients to check for high cholesterol and diabetes. If test results show high cholesterol or indicate that you may have diabetes, your GP can be notified and arrange further investigations and provide any recommended treatment.

17) **Would you be happy to have your cholesterol checked in your dental practice?**

Please circle one answer.

Yes   No   Unsure

18) **Would you be happy to have a diabetes check in your dental practice?**

Please circle one answer.

Yes No Unsure

Section 5: Summary questions

19) **Overall, how important do you believe it is for the dental team to discuss weight and support the public in finding solutions to help the public manage their weight?**

Please add any suggestions or comments here:

20) **Overall, do you think it is a good idea for dental teams to discuss weight with patients and offer referral and / or support to assist with weight management and / or weight loss?**

Please add any suggestions or comments here:

21) **Please add any other comments you feel are relevant in the box below:**

If you are a parent or guardian, please go to Question 22. If you are NOT a parent or guardian, this is the end of the questionnaire. Thank you for your participation.

Section 6: Parent / guardian

If you are a parent or guardian please complete this section.

Please complete the following questions for each child under 18 years of age you have parental responsibility for. Please enter details separately for each child up to a maximum of two children (i.e. Child 1, Child 2). There are four questions for each child which are repeated.

Once you have finished entering your child(ren)'s details, please go to Question 30.

Child 1

22) How old is your child? (years)

Please enter your child's age in the box below:

☐

23) Which of the following best describes your child's sex?

Please circle one answer.

Male   Female   Intersex   Prefer not to say

24) When was the last time your child went to the dentist for a check-up?

Please circle one answer.

In the past 12 months   In the past 1-2 years   Over 2 years ago   Never

25) Are you concerned your child is living with overweight or obesity?

Please circle one answer.

Yes, a lot   Yes, somewhat   Yes, a bit   No   Unsure

Child 2

26) How old is your child? (years)

Please enter your child's age in the box below:

☐

…continued Child 2

27) Which of the following best describes your child's sex?

Please circle one answer.

Male Female Intersex Prefer not to say

28) When was the last time your child went to the dentist for a check-up?

Please circle one answer.

In the past 12 months In the past 1-2 years Over 2 years ago Never

29) Are you concerned your child is living with overweight or obesity?

Please circle one answer.

Yes, a lot Yes, somewhat Yes, a bit No Unsure

30) Research has shown that children living with overweight or obesity are more likely to have dental decay. Considering this information, would you feel comfortable with your child[ren]'s weight and height being measured at a dental appointment?

Please circle one answer.

Yes, definitely Yes, probably Maybe

Probably not Definitely not Don't know

If you have answered 'Yes, definitely', 'Yes, probably' or 'Maybe' please go to Question 32.

If you have answered 'Probably not', 'Definitely not' or 'Don't know' please go to Question 31.

31) Why would you be unsure or not want your child[ren]'s weight and height taken at a dental appointment? Please tick up to three answers which best describe why.

	Tick
My child is in good health and does not have weight-related health problems	
There are more important dental / health issues to discuss	
I do not feel comfortable / my child's weight is private	
I believe it is my responsibility to manage my child's weight	
My child may be treated differently by the dental team if they know their weight	
There is nothing my dentist can do to help my child manage their weight	
Weight should be discussed by another health care professional such as my child's GP	
It may have a negative impact on my child such as them developing negative feelings towards their body image and self esteem	
Other *(please add suggestions or comments here):*	

32) If you wanted to help your child[ren] grow into a healthy weight, would you find it acceptable for your dental team to offer the following support?

Please tick 'Yes' 'No' OR 'Unsure' for each option.

	Yes	No	Unsure
Information about weight management programmes in your local area.			
Referral to weight management programmes in your area with your permission.			
For the dentist to ask your child's GP / practice nurse to discuss your child's weight with you and offer support.			
For the dentist to ask your child's health visitor or school nurse to discuss your child's weight with you and offer support.			
A separate appointment at the dentist to discuss support.			
Information about online resources or mobile applications (apps) that could support your child / family.			

If you have any suggestions of your own, please add them here:

Section 7: Information about your past experiences

If you are a parent / guardian please complete this final section.

33) Have any of the children you have parental responsibility for ever had their weight and height recorded by a dental team?

Please circle one answer.

Yes No Unsure

34) Have you ever been informed by a dentist or a member of the dental team that your child is living with overweight or obesity?

Please circle one answer.

Yes No Unsure

If you have answered 'Yes' or 'Unsure' please answer Question 35.

35) Was any advice or support offered by the dental team following your discussion of your child(ren)'s weight?

Please circle one answer.

Yes No Unsure

If 'Yes' please provide any details here you are happy to share:

This is the end of the questionnaire.

Thank you for your participation.

Figure S2: Summary table to show common themes shared by participants on the importance of dental teams discussing weight and supporting the public in finding solutions to help the public manage their weight

Question: 'Overall, how important do you believe it is for the dental team to discuss weight and support the public in finding solutions to help the public manage their weight?'

Theme	Example quotations	Number of participants to mention theme
**Scope of practice**<br><br>*Views for and against that weight screening / discussion / offer of support is or could be part of the role of an oral healthcare professional*	"I don't think it should be a dentists job to support weight/discuss weight management… No I think that should be down to a GP".<br><br>"I wouldn't have normally put this practice in this area of knowledge and expertise but if it can help patients then that's great".<br><br>"I don't think it is important. Diet is one thing - sugar levels etc, but weight management is not something I think a dentist should be focusing on."<br><br>"Not usually their place, but an interesting idea of integrating this into dental care - which we know is important for other aspects of health. So, I'm supportive and think it's important."<br><br>"I think it is important for the dental team to be involved but they would need appropriate training to broach what can be a very sensitive subject".<br><br>"Any opportunistic intervention would be useful, however I would say in a dental setting smoking and alcohol would be more immediately relevant."<br><br>"I think dental teams are best placed to provide advice as they can link up with other medical teams"	625

	"I think it is a good idea. I feel it would be appropriate for such info to be carried out by dental assistant nurse etc."<br><br>"Yes they are trained professionals we have faith and trust in".<br><br>"Yes patients might feel more comfortable and be more open talking to the dentist."	
**Sensitive nature of intervention**<br><br>*Views that weight screening / discussion with patients could make patients feel uncomfortable and potentially lead to some patients avoiding attending dental services. Risk of inducing weight stigma raised.*	"It would be an added service that I would find acceptable, but understand that a lot of people might find it intrusive".<br><br>"No I would be highly offended if a dentist spoke about weight management".<br><br>"This is a nice idea so long as it does not deter patients from going to the dentist if they may be embarassed about their weight".<br><br>"Yes. The only negative I can see is that it may be an awkward topic to raise for the dentist, as the patient would not necessarily expect to be asked these questions."<br><br>"I think this could affect mental health especially in teenagers if they are being told they are overweight in every health appointment".<br><br>"I don't think it's helpful - if weight is an issue for people or they have a history of eating disorders it may cause added stress or be upsetting."<br><br>"Asking about weight could be seen as offensive and lead to discrimination."<br><br>"I understand weight management is important and if handled in a sensitive way it could be effective at dental practices BUT it has to be handled sensitively."<br><br>"Addressing obesity is very important but I feel this is the wrong intervention. I think we risk putting people off getting regular care. The last thing most overweight people need is yet one more person telling them they should cycle to work."	44

**Duplication of efforts**<br><br>*Participant views that management of weight can already be accessed through other resources or services or managed by an individual independently meaning dental involvement is not required or important.*	"Fairly [important], but the doctor does this too / Maybe if the doctor hasn't mentioned it".<br><br>"I have Type 2 diabetes and am currently under my GP. I don't know if I would benefit if my dentist took over monitoring as its related to my diagnosis and I may need medication adjustments."<br><br>"Annual health check at GP Surgery covers these issues."<br><br>"It may be a good idea but I don't believe it would make any difference because people can access those information online."<br><br>"Not important to me maybe others would benefit".<br><br>"It's quite important but although I am overweight I am hardly ever ill and not under hospital"<br><br>"I'm not saying it's a bad idea - just not for me"<br><br>"I am fit and healthy and don't think I would benefit from this service."<br><br>"Someone has to, it's important and people don't realise they are overweight".<br><br>"I have scales and know my height so can work out my own BMI. I don't feel it necessary to have this done my a dentist".<br><br>"For me personally I don't think it's relevant as I self manage. For others it could be helpful and I wouldn't want to obstruct that pathway if it could be useful to some."<br><br>"not very [important], I feel like most people are aware of risks and seek help independently"	181

**New concept**<br><br>*Views shared by participants that this is a novel proposal and express uncertainty over such an intervention.*	"feels strange".<br><br>"I've never thought of this before (or made the connection) but it sounds like a good idea".<br><br>"Unsure it is a new concept but im sure it could become the norm".<br><br>"People would get used to this being by a dentist not a doctor so it would be important. I get weighed weekly and have support in this area at the moment."<br><br>"Find it difficult to understand why dental teams would get involved. Perhaps because it is a new concept that I haven't previously thought about".	37
**Lack of time and resources for oral health care teams to implement**<br><br>*Views shared by participants that dental teams do not have the time or resources to provide weight screening and supportive interventions. If implemented, concerns raised over the impact this may have on reducing access to dental services.*	"probably not as dentist are busy, I prefer they spend time to sort out dental problems of their patients".<br><br>"There are not enough NHS dentists/practices for existing number of patients. Please don't waste their time on non dental appointments. Use other professionals if this type of service is being considered at dental practices."<br><br>"great idea if they have time".<br><br>"In an ideal world of funding the NHS would offer a fully integrated service of dental and medical help for patients. In my current experience lack of NHS Dentists due to current government policy (2023) and possible loss of our NHS system puts this all in question. This system would work well where NHS Dental Services are in GP / medical centres and patients used to integrated services."<br><br>"I think its important to discuss weight management but as above I am not sure a dental practice/surgery is the right place. I believe its important but I am sure if the dental team would have the time and knowledge to carry this out without detriment to either dental treatment or weight management."	127

**Criteria for an acceptable service in dental settings**<br><br>*Suggestions by participants on acceptable approaches to be undertaken by dental teams or proposed criteria to increase acceptance by the wider public including:*<br>• *Patient led weight screening / discussion*<br>• *Voluntary service*<br>• *Patients to be informed of weight screening / offer of intervention prior to appointment*<br>• *Sensitive communication*<br>• *Use of posters/leaflets and brief intervention approach*<br>• *Link to oral health*	"If the patient wants help and discloses this when talking about the need to reduce sugary drinks and snacks for dental health, then yes but otherwise no."<br><br>"I think if a patient requests such support from the Dentist, then it is appropriate."<br><br>"Put up signs saying they can, but don't start onto heavier people unless they initiate."<br><br>"Only if the individual asks for it you do not want to put people off going to the dentist"<br><br>"Only if the person giving advice isnt overweight and can lead by example"<br><br>"Only if they are appropriately trained/qualified and not overweight themselves."<br>"Would be good - take Strain off GP's however would want informing prior to appointment, I think as not everyone is comfortable with weight etc".<br><br>"Yes should be routine and expected".<br><br>"I think the approach should be more about healthy eating as it affects dental health as well as overall health"<br><br>"More important for healthy lifestyle to be discussed rather than weight"<br><br>"Obesity needs to be a mtd approach, but I would not want a dentist time to be taken up too much, only give brief intervention and refer on."<br><br>"I think the dental team have enough going on without adding to their work load, information and leaflets would be fine."<br><br>"[do you think it is a good idea…]No, but provision of other resources such as leaflets might be a good idea"	47

	“The clinical dental team is already very time strapped treating NHS patients, an auxiliary/HCA could do this role. All health professionals should carry out brief health interventions. Webs sites could have interactive areas patients could enter height, weight, log diet etc. Get information re diet exercise, self refer to weight management healthy lifestyles . Perhaps a text email invite to patients would reduce the burden of screening a large number. Weight needs to be chalked in a tactful manner, I find measuring the waist with a BMI taper measure is non threatening, showing the patient the colour and asking them what the feel about the yellow or red colour displayed. This approach starts a good engagement to then discuss ways forward re weight loss, diet exercise etc.”<br><br>“May be relevant to make patients more aware of the link between health/diet and dental health”<br><br>“I would be happier if they relate it to dental and oral issues”.<br><br>“Potentially but I think there would need to be considerable PR to prepare the public for this”.<br><br>“If requested, but I wouldn't want this to be a 'forced' requirement of going to the dentist.”<br><br>“Yes, as with a GP, but consent should be obtained before any discussions or measurements”<br><br>“Yes - as long as they are approachable and have excellent communication skills which are monitored.”<br><br>“I think any support offered would be welcome as long as it is handled in a sensitive way”<br><br>“Yes as long as it was at no extra charge, because I pay for check ups so I would not be prepared to pay for others”.	

**Recognition of a holistic approach**<br><br>*Participants sharing views that all healthcare professionals have a duty to support the public with weight and health, including dental teams, and that this could be beneficial for the public.*	"Very important as it will help with overall health and wellbeing".<br><br>"I believe this should be a responsibility of every member / service within the health service: "Make Every Contact Count"".<br><br>"Any supportive intervention in helping to resolve this important medical and social issue is to be welcomed. The emphasis should be on supportive."<br><br>"Very important good health is important and a holistic approach. One stop shop would be helpful for busy people".<br><br>"I believe any health care profession should be able to help maintain weight management. If they had the right training & knowledge to do it, they should be able to help anyone who is struggling."<br><br>"Overall I think it is a good idea If I am already attending the appointment I may as well link dental hygiene to weight".<br><br>"Any health care service should provide health improvement service, advice & ongoing referral options".<br><br>"Every opportunity to check is surely a good thing - so more opportunity means better & quicker help".<br><br>"To me this is crucial to all health needs. There needs to be a link between health care weight management and the well being of our teeth management. 100% this is essential. I think with this future dentistry need will be better managed".<br><br>"Yes the more its talked about the better / Yes the more support the better".<br><br>It is important for all healthcare providers to work collaboratively in managing the health of their patients. "Sometimes little details of significance can be picked at the dentist…it	264

	is important to evaluate the health of the patient in general e.g. mental health. Looking at the root cause of "binge" eating or drinking maybe caused by mental health."<br><br>"Very important but need to work in a coordinated manner. Pharmacies could also be proactive."<br><br>"It is a start, but many of the solutions to the obesity epidemic lie with governments and businesses, such as taxing high-sugar, heavily processed foods."	
**Association between oral and general health**<br><br>*Contrasting views shared by participants on whether oral and general health are associated reinforcing the importance and relevance of dental team support with weight versus participants who view oral health as being separate from general health and weight and as such should be managed by different healthcare teams.*	"very important because diet affects teeth as well as overall health"<br><br>"The links with a high sugar diet between weight & dental issues are well known so I do think it is a good idea for dental teams to be involved".<br><br>"Good health helps with oral health and would benefit both patient and medical practice"<br><br>"I find it all contributes from weight to diet to foods eaten to your health of your teeth & gums"<br><br>"Very important , physical health, mental health and dentistry should all be linked as need all to be healthy to have healthy happy life".<br><br>"As there are links between oral health and other conditions such as heart disease which can also be impacted on by weight it's very important."<br><br>"There's a direct correlation between tooth decay and poor diet, so it's important to our overall well-being to maintain a healthy weight."<br><br>"Eating the wrong food affects teeth, weight, general health - makes perfect sense as most people have a regular interaction with dentist but might not see GP for years if not unwell."<br><br>"Unless it is teeth or gum related I don't think that it is important and should be dealt	126

	with by a persons GP".<br><br>"I guess its important, though I don't quite understand how it relates to dental hygiene."<br><br>"…most patients probably wouldn't see the link. You might need to start with a program to link what you eat with dental health."<br><br>"Not sure. I think if it's clear that the discussions regarding weight are linked to oral health in some way, otherwise what is the reason."<br><br>"Only relevant if a patient's diet is causing dental decay, so advice should be given on which foods and drinks to avoid."<br><br>"I think their key strength is in supporting overweight children by helping parents understand the role of food in dental problems AND weight. I don't support having more health professionals pester adults."	
**Improving access to weight support**<br><br>*Participant views that weight screening and support offered by dental teams could improve access to weight management services with the potential to benefit more people.*	"hard to see anyone at the moment, so it would help".<br><br>"because of the check-up appointments & believe dentist see patients on a more regular basis than doctors & can notice & observe weight gains & changes to a patients body size".<br><br>"Yes may be the only contact with medical professional that someone has."<br><br>"It is important if the dentist is your only source".<br><br>"important as we tend to see the GP less and less and they might have more time to address these issues".<br><br>"very important if someone needs support and the NHS list is very long they may get support quicker this way"	61

	"Important for people to have more opportunities to look after themselves and get help where needed".<br><br>"Just for overall well being - I actually see dentist more often than GP".<br><br>"I think it is important there doesn't seem to be much support in the community & can't get appts with GP"<br><br>"I think it is important as it may not get picked up anywhere else. I very rarely go to the doctors so if I had high cholesterol or diabetes I would like to know."<br><br>"Yes. Dental and Optician checkups are the only routine regular interactions which most of us have with medical professionals".<br><br>"Perhaps other health professionals would be better, and based in locations available to more of the target groups".<br><br>"I think it is important that people can get this support readily. If they attend a dentist regularly for check-ups, this would seem a reasonable source of such advice or support assuming dentists are appropriately trained".	

Figure S3: STROBE Statement—Checklist of items that should be included in reports of *cross-sectional studies*

	Item No	Recommendation	Page No
**Title and abstract**	1	(*a*) Indicate the study's design with a commonly used term in the title or the abstract	1
		(*b*) Provide in the abstract an informative and balanced summary of what was done and what was found	3
**Introduction**			
Background/rationale	2	Explain the scientific background and rationale for the investigation being reported	4-5
Objectives	3	State specific objectives, including any prespecified hypotheses	5
**Methods**			
Study design	4	Present key elements of study design early in the paper	5-6
Setting	5	Describe the setting, locations, and relevant dates, including periods of recruitment, exposure, follow-up, and data collection	5-8
Participants	6	(*a*) Give the eligibility criteria, and the sources and methods of selection of participants	6
Variables	7	Clearly define all outcomes, exposures, predictors, potential confounders, and effect modifiers. Give diagnostic criteria, if applicable	7-8
Data sources/ measurement	8*	For each variable of interest, give sources of data and details of methods of assessment (measurement). Describe comparability of assessment methods if there is more than one group	*7-8*
Bias	9	Describe any efforts to address potential sources of bias	7-8
Study size	10	Explain how the study size was arrived at	7
Quantitative variables	11	Explain how quantitative variables were handled in the analyses. If applicable, describe which groupings were chosen and why	7-8
Statistical methods	12	(*a*) Describe all statistical methods, including those used to control for confounding	7-8
		(*b*) Describe any methods used to examine subgroups and interactions	7-8
		(*c*) Explain how missing data were addressed	8
		(*d*) If applicable, describe analytical methods taking account of sampling strategy	7-8
		(*e*) Describe any sensitivity analyses	N/A
**Results**			
Participants	13*	(a) Report numbers of individuals at each stage of study—eg numbers potentially eligible, examined for eligibility, confirmed eligible, included in the study, completing follow-up, and analysed	8-9
		(b) Give reasons for non-participation at each stage	8
		(c) Consider use of a flow diagram	N/A
Descriptive data	14*	(a) Give characteristics of study participants (eg demographic, clinical, social) and information on exposures and potential confounders	Table 1
		(b) Indicate number of participants with missing data for each variable of interest	8

Outcome data	15*	Report numbers of outcome events or summary measures	8-9
Main results	16	(*a*) Give unadjusted estimates and, if applicable, confounder-adjusted estimates and their precision (eg, 95% confidence interval). Make clear which confounders were adjusted for and why they were included	Pages 8-12. Tables 2, 3, 4.
		(*b*) Report category boundaries when continuous variables were categorized	Tables 2, 3, 4.
		(*c*) If relevant, consider translating estimates of relative risk into absolute risk for a meaningful time period	n/a
Other analyses	17	Report other analyses done—eg analyses of subgroups and interactions, and sensitivity analyses	12-14
**Discussion**			
Key results	18	Summarise key results with reference to study objectives	14-16
Limitations	19	Discuss limitations of the study, taking into account sources of potential bias or imprecision. Discuss both direction and magnitude of any potential bias	17
Interpretation	20	Give a cautious overall interpretation of results considering objectives, limitations, multiplicity of analyses, results from similar studies, and other relevant evidence	14-18
Generalisability	21	Discuss the generalisability (external validity) of the study results	14-18
**Other information**			
Funding	22	Give the source of funding and the role of the funders for the present study and, if applicable, for the original study on which the present article is based	1

*Give information separately for exposed and unexposed groups.

Note: An Explanation and Elaboration article discusses each checklist item and gives methodological background and published examples of transparent reporting. The STROBE checklist is best used in conjunction with this article (freely available on the Web sites of PLoS Medicine at http://www.plosmedicine.org/, Annals of Internal Medicine at http://www.annals.org/, and Epidemiology at http://www.epidem.com/). Information on the STROBE Initiative is available at www.strobe-statement.org.
